# Investigation of the effects of propofol/ketamine versus propofol/fentanyl on nausea-vomiting administered for sedation in children undergoing magnetic resonance imaging: a prospective randomized double-blinded study

**DOI:** 10.3906/sag-2009-98

**Published:** 2021-08-30

**Authors:** Hacı Semih GÜRCAN, Ayşe ÜLGEY, Özlem ÖZ GERGİN, Sibel SEÇKİN PEHLİVAN, Karamehmet YILDIZ

**Affiliations:** 1 Department of Anesthesiology and Reanimation, Gümüşhacıköy Government Hospital, Amasya Turkey; 2 Department of Anesthesiology and Reanimation, Faculty of Medicine, Erciyes University, Kayseri Turkey

**Keywords:** Magnetic resonance imaging, child, ketamine, fentanyl, deep sedation, propofol

## Abstract

**Background/aim:**

In this study, we aimed to compare the effects of propofol-ketamine and propofol-fentanyl sedations on post-procedure nausea-vomiting in children undergoing magnetic resonance imaging (MRI).

**Materials and methods:**

This study included 100 pediatric patients (2–10 years old) who had propofol-ketamine and propofol-fentanyl for sedation to undergo MRI. The patients were divided into two groups, and sedation was performed through propofol-ketamine (Group K; n = 50) or propofol-fentanyl (Group F; n = 50). For sedation induction, intravenous (IV) bolus of 1.2 mg/kg propofol and 1 mg/kg ketamine were administered in Group K, IV bolus of 1.2 mg/kg propofol, and 1 µg/kg fentanyl in Group F. All patients received 0.5 mg/kg IV bolus propofol in additional doses when the Ramsay Sedation Score (RSS) was below 4 for maintenance. Perioperative heart rate, systolic arterial pressure, peripheral oxygen saturation, respiratory rate, and nausea-vomiting scores were recorded for each patient.

**Results:**

There was no difference between the groups in terms of nausea incidences at the 1st hour. However, the rate of vomiting was significantly higher in Group K.

**Conclusion:**

In our study, we showed that the vomiting rate was higher in the 1st hour in Group K compared to Group F.

## 1. Introduction

Magnetic resonance imaging (MRI) system is a procedure that requires patients to remain motionless for a long time in a claustrophobic and noisy environment. Sedation should be performed on pediatric patients during imaging as pediatric patients cannot remain still due to severe anxiety. In anesthesia applications performed only for imaging purposes, conscious sedation, deep sedation, total intravenous anesthesia (TIVA), or inhalation anesthesia can be performed [1,2].

An essential point in examinations for pediatric patients in MRI units is to increase the patient circulation rate without compromising patient safety. For this reason, combinations, of which the effect of which starts quickly and ends quickly, allowing the shortening of discharge time, are crucial.

The effects of ketamine can be listed as sedation, hypnosis, dissociation, analgesia, and amnesia. The anesthetized state has been termed dissociative anesthesia because patients who receive ketamine alone appear to be in a cataleptic state, in contrast with other states of anesthesia that resemble normal sleep. Ketamine increases systolic arterial pressure, heart rate, and cardiac output in a biphasic manner. It produces a direct cardiodepressive, negative inotropic effect next to an indirect stimulatory effect due to activation of the sympathetic system [3].

Fentanyl is currently the most widely used drug as the analgesic component of balanced anesthesia. It is a synthetic opioid agonist, a potent narcotic analgesic and has the same characteristics as other opioids. That is, it causes analgesia, sedation, respiratory depression, and nausea, vomiting [4]. The effect of fentanyl on the cardiovascular system is minimal. Cholinergic effects such as nausea, vomiting, myositis, and constipation may be seen [5].

Propofol is used only intravenously. The onset of the effect is fast, and the duration is short. It is used in conscious sedation, general anesthesia induction, and maintenance. It does not cause a permanent effect after anesthesia. The use of propofol outside the operating room is gradually increasing. The reason for this is that it is easy to use, effective, and has a safe profile. However, it also has several other advantages, such as the rapid onset of effect, rapid metabolism, rapid separation, and showing antiemetic activity [6,7]. Since propofol is hypnotic with no analgesic effect, it is recommended to be used with ketamine or a short-acting opioid in daily practice. The combination of propofol and ketamine has gained popularity in short-time procedures to provide sedo-analgesia [8].

Postoperative nausea and vomiting (PONV) risk continue to be an essential problem for patients due to anesthetic methods and drugs. Nausea can be experienced alone or with vomiting. If airway reflexes are depressed because of the residual effects of anesthetic and analgesic drugs, pulmonary aspiration risk because of vomiting is high. Also, persistent vomiting may cause dehydration and electrolyte imbalance. It may delay the discharge of the patient, especially after daily procedures [9,10,11].

In this study, we aimed to evaluate whether there is a difference in terms of nausea and vomiting between propofol/fentanyl and propofol/ketamine combination, which are two of the routine methods used in our clinic in the sedation of patients in the pediatric age group (2–10 years).

## 2. Materials and methods

**Figure 1:**
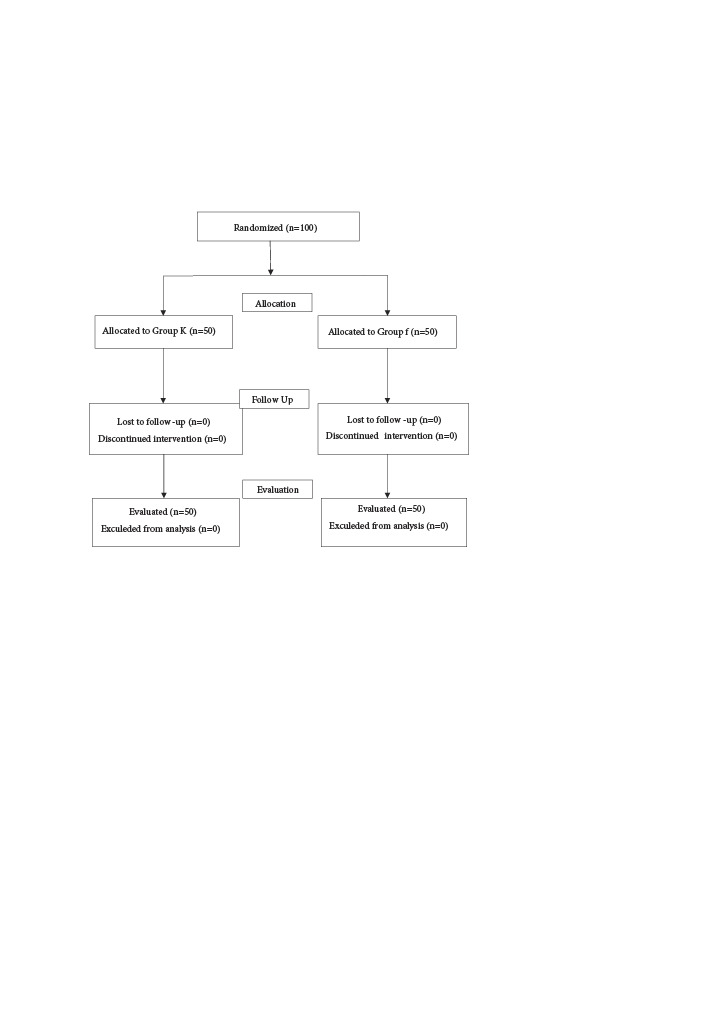
CONSORT flow diagram of the study.

This study was carried out with 100 pediatric patients in between 2 to 10 years old who underwent imaging in the Erciyes University Faculty of Medicine, Department of Anesthesiology and Reanimation, Erciyes University, Gevher Nesibe Hospital MRI unit for diagnostic purposes. After Faculty Ethics Committee approval (Decision Number: 2017 / 285) and informed consent forms from the families of these patients were obtained, these patients were included in the study as prospective randomized double-blind. Tosun et al. have reported the incidence of PONV in children undergoing strabismus surgery as 60% in their study. With respect to that study, using α = 0.05 and β = 0.2 for each comparison, the sample size in the current study was estimated at 48 evaluable patients per group [12]. Patients who were ASA physical status I or II were enrolled into the study, and the patients who had a severe hemodynamic problem (using an inotropic-vasoactive agent), partial loss of consciousness or were in a coma, who was found to have upper respiratory tract infection at the time of imaging, who had an intracranial space-occupying lesion, organ failure, who was suspected of non-adherence to the duration of fasting and had tonsillar hyperplasia causing airway obstruction were planned to exclude from the study (CONSORT flow diagram: Figure 1). 

All patients were prevented from taking solid food 6 h before and liquid food 2 h before anesthesia.

The existing comorbidities of the patient were determined, and the drugs used by the patient and their interaction with anesthetic drugs were evaluated.

After establishing the vascular access, providing premedication with intravenous (IV) 0.05 mg/kg midazolam, they were taken to the MRI room with their parents 30 min before the MRI imaging process started, and the panic and fear that children felt due to a foreign environment were tried to be avoided. In order to avoid any kind of bias, 1:1 block randomization was performed. Clinical and study staff involved in recruitment, sedation, or patient care, children and their parents remained blinded until observation of the last patient completed. An anesthetist, who was not involved in patient care prepared the study medications.

Patients in group K, following IV bolus of 1.2 mg/kg propofol and 1mg/kg ketamine application, received 0.5 mg/kg IV bolus propofol in additional doses when the Ramsay sedation score (RSS) was <4 for maintenance. Administration of atropine (0.015 mg/kg IV) was planned in case of the probability of hypersecretion due to ketamine.

After IV bolus of 1.2 mg/kg propofol and 1 µgr/kg fentanyl administration, patients in group F received 0.5 mg/kg IV bolus propofol in additional doses when the RSS was <4 for maintenance.

Heart rate (HR), systolic arterial pressure (SAP), diastolic arterial pressure (DAP), mean arterial pressure (MAP), peripheral arterial oxygen saturation (SPO_2_) of the patients were monitored throughout the procedure, and pre-induction baseline values, 10th, 20^th^ min and the 1st h values were recorded.

Holding breath that lasted more than 20 s, although head tilt-chin lift maneuver was maintained, or being unable to breathe was considered as apnea. Children under SP02 value of (90%) were determined to develop hypoxia and desaturation, and tactile stimulation and airway opening maneuvers were performed. When there were coughing and suspicion of airway obstruction, the imaging was interrupted, and the airway patency was checked after pulling the patient out of the magnetic field. If the airway obstruction was partial, a position to provide head tilt was given. Overall, it was planned to ventilate with mask-ambu, and place a laryngeal mask airway (LMA), if necessary, and apply orotracheal intubation in case of failure.

After completing the imaging, patients who were taken to the recovery room were kept under monitoring, and the hemodynamics, respiratory, and consciousness status of the patients were followed. It was observed whether there were nausea-vomiting and agitation. The patients were followed up in the recovery unit for 2 h, and the patients who were recovered were discharged after their parents were asked to observe the patients for 24 h for nausea and vomiting. After 24 h, the parents were called, and the 12- and 24-h results were recorded. Pre-procedure, 1st-hour, 12th-hour, and 24th-hour nausea-vomiting scores were recorded using a numeric scoring system for PONV [13]. (Table 1).

**Table 1:** Postoperative nausea vomiting score.

Postoperative nausea vomiting score	
0	No vomiting
1	Nausea is present, no vomiting
2	Vomiting once in 30 min
3	Two or more vomiting in 30 min

Modified Aldrete scoring was used to evaluate patients’ recovery [14]. (Table 2).

**Table 2:** Modified Aldrete scoring.

Modified Aldrete scoring	Score value
OXYGENATIONSpO2> 92% in room airSpO2< 90% with oxygen supportSpO2< 90% with oxygen support	210
Breathes deeply and coughs comfortablyDyspneic, superficial, or limited breathingApnea	210
Blood pressure ± 20 mmHg of normalBlood pressure ± 20-50 mmHg of normalBlood pressure ± 50 mmHg of normal	210
Completely awakenedCan be awakened by verbal warningsUnresponsive	210

The time to modified Aldrete scoring ≥9 was recorded as recovery time.

IBM SPSS Statistics 22.0 software package (IBM Corp., Armonk, NY, USA) was used for statistical analysis of the data. Independent student t-test was used to compare heart rate, Mann–Whitney U test was used to compare systolic and diastolic arterial pressure, duration of the procedure, propofol doses, and Chi-square test was used for postop nausea and vomiting score analysis. The compliance of the data to normal distribution was evaluated by histogram, q-q graphs and Shapiro–Wilk test. Variance homogeneity was tested with the Levene test. The p value <0.05 was considered statistically significant.

## 3. Results

When the demographic data of the patients were evaluated, no statistically significant difference was found between the two groups in terms of age, weight, propofol amount, and duration of the procedure (p > 0.05) (Table 3).

**Table 3:** Demographic data of the patients: Mann–Whitney U test (p < 0.05).

	Group F(n = 50)	Group K(n = 50)	p
Age (years)	5 (2–10)	4 (2–10)	0.258
Weight (kg)	17.50 (10–50)	17 (10–30)	0.857
Propofol amount (mg)	22 (12–134)	24 (12–76)	0.885
Duration of Procedure (min)	24 (9–45)	21.50 (13–58)	0.392
Recovery time (min)	55 (28–90)	55 (13–75)	0.736

**Figure 2:**
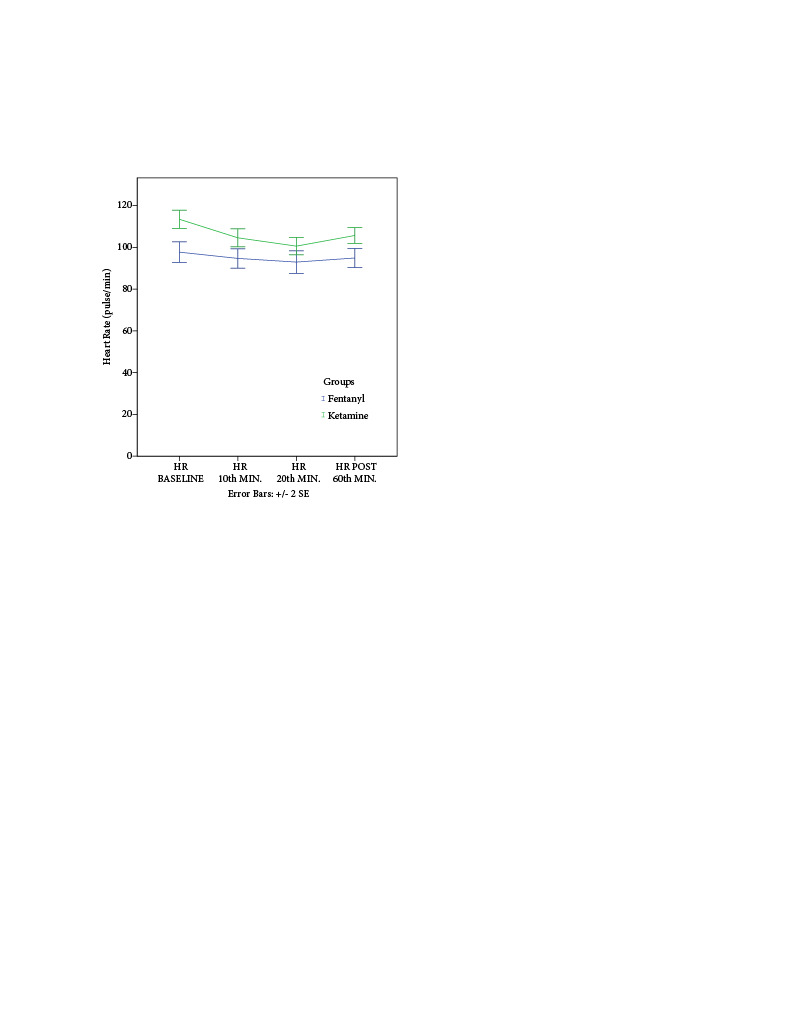
Comparison of heart rates: independent student t-test (p < 0.05).

When the patients’ HR was evaluated, no significant difference was found between Group F and Group K in the baseline, 10th-min, 20th-min, and postoperative 60th-min data (p > 0.05) (Figure 2).

**Figure 3:**
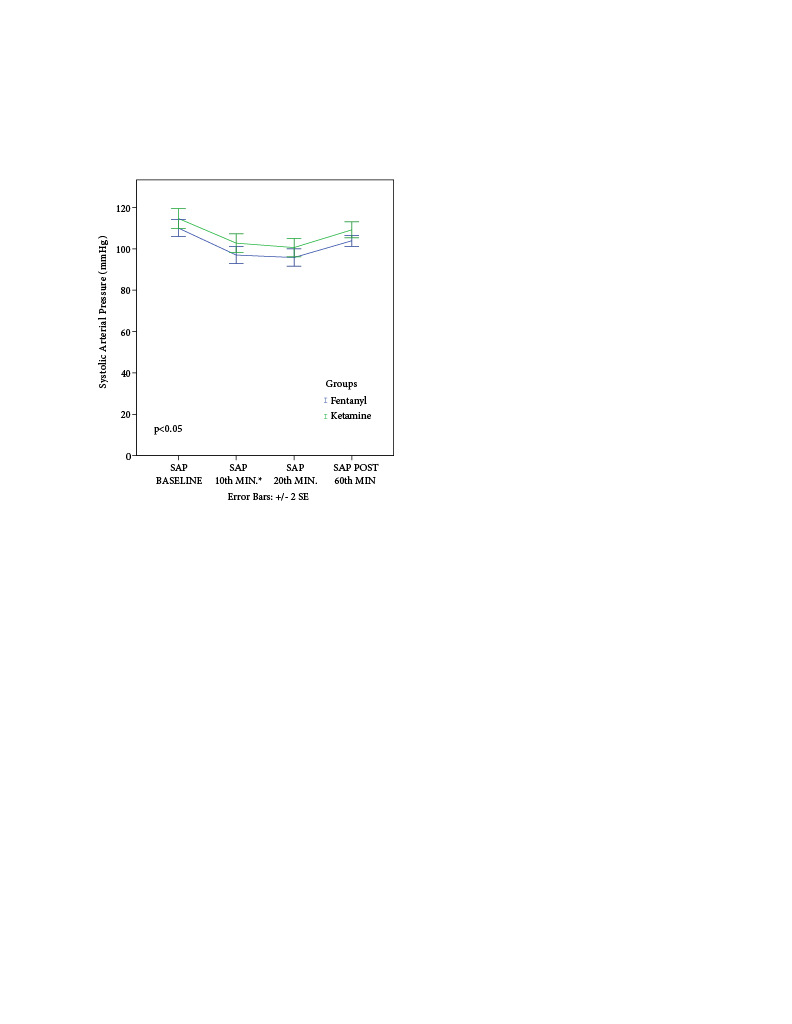
Comparison of systolic arterial pressures: Mann–Whitney U test (p < 0.05).

When SAP of patients was evaluated, no statistically significant difference was found between the two groups in the baseline, 10th min, 20th min, and 60th-min postoperative data (p > 0.05) (Figure 3).

**Figure 4:**
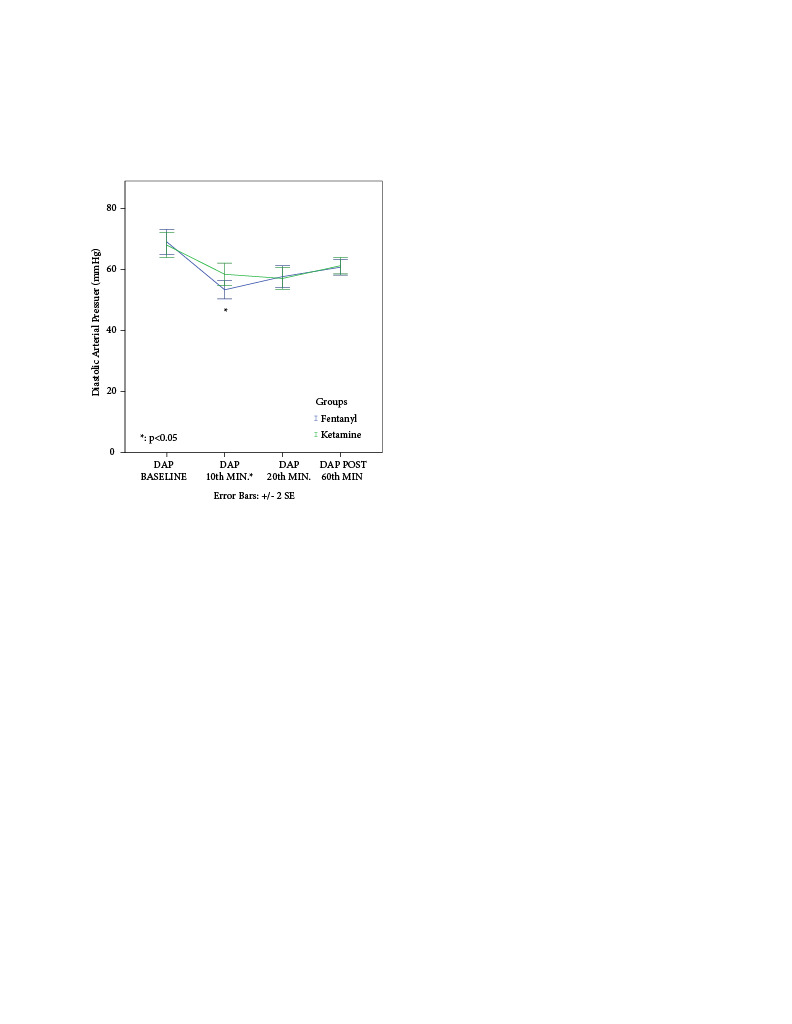
Comparison of diastolic arterial pressures: Mann–Whitney U test (p < 0.05).

When DAP of patients was evaluated, while no significant difference was found between Group F and Group K in the baseline, 20th min, and postoperative 60th-min data (p > 0.05), a significant difference was found in 10th-min data (p < 0.05) (Figure 4).

When postoperative nausea and vomiting scores of the patients were evaluated, there was no difference between the groups in terms of nausea rates at the 1st h (In Group K vomiting was observed once in six patients while it was not observed in Group F at the 1st h). At other hours, no significant difference was observed between the groups in terms of nausea-vomiting rates (p > 0.05) (Table 4).

**Table 4:** Postoperative nausea-vomiting scores (PONVS) of the groups: Chi-Square test (p < 0.05).

	PONV scores	Group F(n = 50)	Group K(n = 50)	Comparisons
PONVS BASELINE	0	48 (96%)	45 (93%)	Χ2=1.382p = 0.436
	1 and -	2 (4%)	5(10%)	
PONVS 1ST HOUR	0	45 (90%)	44 (88%)	Χ2 = 0.102p = 0.749
	1 and -	5 (10%)	6 (12%)	
PONVS 12TH HOUR	0	47 (94%)	48 (96%)	Χ2=0.211p = 1.000
	1 and -	3 (6%)	2 (4%)	
PONVS 24TH HOUR	0	50 (100%)	49 (98%)	Χ2=1.010p = 1.000
	1 and -	0 (0%)	1 (2%)	

## 4. Discussion

This study aimed to compare the effects of propofol-ketamine versus propofol-fentanyl sedations on post-procedure nausea-vomiting in children undergoing MRI. The main results were the significantly higher vomiting rate at the 1st h and significantly higher DAP at the 10th minute in Group K. 

PONV has been described after ketamine administration [15]. Green et al. compiled approximately 100 studies that ketamine was applied. They found that more than 11,000 patients had vomiting at a rate of 8.5%, and that vomiting was in the late recovery stages where patients generally began to wake up [16]. In the application of fentanyl, PONV has been described too [17], and concerns exist that this may also be true in combination with propofol. In our study, PONV incidences were low, which may be due to the antiemetic effect of propofol [18,19,20].

Vomiting was seen in 6 (%12) patients in the 1sthour in Group K, but none of them required rescue medication because they vomited once within 30min. Vomiting was not observed in Group F in the 1st hour. Godambe et al. compared the effectiveness of propofol-fentanyl and ketamine-midazolam for brief orthopedic procedural sedation in 113 pediatric patients. They also observed no vomiting in the propofol-fentanyl group [21]. Bauman et al. randomly chose 64 of the total of 243 sedation procedures with analgesia for a descriptive retrospective review and analysis in pediatric patients. They reported no nausea and vomiting in the propofol-fentanyl groups. [22]

Atropine, which increases HR with minimal effects on mean arterial pressure (MAP) and cardiac output (CO), is a competitive antagonist of cholinergic receptors. Atropine administration also results in a decrease in the incidence of postoperative nausea and vomiting [23]. In case of hypersecretion due to ketamine, atropine administration was planned, but none of the patients experienced hypersecretion that would require atropine administration.

Green et al. detected vomiting in 12.1% of cases above five years of age and 3.5% of cases under five years of age after ketamine administration [24]. This study shows that vomiting after ketamine administration may be associated with increased age. In our study, no statistically significant difference was found between the two groups in terms of the age of the patients.

When the hemodynamic parameters of the patients were evaluated, no statistically significant difference was found between the two groups in HR and SAP in our study; DAP in the 10th-min after sedation administration was found to be significantly higher in the ketamine group than in the fentanyl group. However, patients in the propofol-fentanyl group had lower HR and SAP than patients in the propofol-ketamine group. We concluded that the hypotensive effect of propofol was balanced with the use of ketamine [25,26,27].

However, Sinner and Graf stated in their study that it is appropriate to use ketamine, especially in cases where the cardiovascular system is unstable [28]. In terms of cardiovascular stability, we can say that ketamine is an appropriate alternative to the risk of propofol-related hemodynamic depression development due to its sympathomimetic effect.

In our study, it was determined that there was no statistically significant difference between the two groups in terms of recovery time.

In conclusion, we showed in our study that there was no difference between the groups in terms of nausea rates; however, the vomiting rate in Group K was higher than Group F within the 1st hour. 

## Informed consent

Ethical approval for this study was obtained from Faculty Ethics Committee (Decision Number: 2017 / 285). Informed consent forms were obtained from the families of patients before the study.
